# Beneficial Effects of Sagacious Confucius' Pillow Elixir on Cognitive Function in Senescence-Accelerated P8 Mice (SAMP8) via the NLRP3/Caspase-1 Pathway

**DOI:** 10.1155/2019/3097923

**Published:** 2019-11-06

**Authors:** Zhitao Hou, Fengjin Li, Jing Chen, Yitian Liu, Changyuan He, Meng Wang, Tingting Mei, Yue Zhang, Liying Song, Xianming Shao

**Affiliations:** ^1^The College of Basic Medical and Sciences, Heilongjiang University of Chinese Medicine, Harbin, Heilongjiang 150040, China; ^2^Heilongjiang Academy of Traditional Chinese Medicine, Harbin, Heilongjiang 150036, China; ^3^Beijing University of Chinese Medicine, Beijing 100029, China

## Abstract

Sagacious Confucius' Pillow Elixir (SCPE) is a traditional Chinese medicine that is mainly used for cognitive impairment in aging; however, the underlying mechanisms remain unclear. Aging is one of the most important pathogenic factors leading to inflammation and pyroptosis in the hippocampus, which may be a potential mechanism in elderly patients with cognitive impairment. Here, we examined whether SCPE could improve cognitive impairment in SAMP8 mice by reducing hippocampal inflammation and pyroptosis. Seven-month-old senescence-accelerated P8 mice (SAMP8) received SCPE (2.3 g/kg/day; 4.6 g/kg/day; 9.2 g/kg/day) for 28 days. Cognitive function and morphometric examinations were performed followed by water maze testing, hematoxylin-eosin staining, Congo red staining, toluidine blue staining, and TUNEL analysis of hippocampal CA1 and CA3 regions. Escape latency increased and times across platforms decreased in SAMP8 mice; however, both of them were normalized by SCPE after 28 days. Aging caused significant pyroptosis in hippocampal CA1 and CA3 regions, as evidenced by neuronal degeneration and necrosis, amyloid deposition, and decreased Nissl body amounts after cognitive impairment, which were greatly improved by SCPE. SCPE reduced serum IL-1*β*, IL-6, IL-18, and TNF-*α* levels and reduced hippocampal NLRP3, ASC, caspase-1, GSDM-D, IL-1*β*, IL-6, IL-18, and A*β* expression. Thus, SCPE exerts an antipyroptotic effect in aging, mainly by suppressing the NLRP3/caspase-1 signaling pathway.

## 1. Introduction

Cognitive impairment (CI) is a common complication of aging [1]. The predicted incidence of CI in people over 60 years old is 1%, and the rate increases to 8% for those older than 65 years. Moreover, the morbidity of the elderly above 85 years old is as high as 20% [2,3]. Currently, CI is the most common and least treatable of all geriatric diseases. Most CI drugs are used for the treatment of Alzheimer's disease (AD); however, their efficacy remains uncertain, and unpleasant side effects limit the application of these drugs, which mainly include cholinesterase inhibitors, glutamate receptor blockers, calcium ion antagonists, and neuron protectants [4–6].

Traditional Chinese medicine has been used to treat CI for thousands of years. Some Chinese medicinal herbs, such as *Polygala tenuifolia* Willd (Chinese name, yuan zhi) [7] and *Acorus tatarinowii* (Chinese name, shi changpu) [8], have significant curative effects in treating almost all kinds of dementia. Among traditional Chinese medicine formulas, Sagacious Confucius' Pillow Elixir (SCPE) [9], Kai-Xin San [10] and decoction of Rehmannia [11] are the most frequently clinically prescribed Chinese medicinal products to treat CI.

SCPE is a classic Chinese medicinal formula mainly used for treating cognitive decline, which was first described in Essential Prescriptions from the Invaluable Prescriptions for Ready Reference (Beiji Qianjinyaofang) by the “king of traditional Chinese medicine” doctor Si-Miao Sun during the Tang Dynasty (581∼682 AD). For improving learning and memory ability, SCPE is the most frequently prescribed clinical Chinese medicinal product. SCPE consists of four herbs: *Acorus tatarinowii*, *Polygala tenuifolia* Willd, *Chinemys reevesii* (*Gray*), and *Fossilia Ossia Mastodi*. The traditional effects of SCPE are tonifying the kidneys, calming nerves, and improving learning and memory ability. SCPE has anti-inflammatory, antiapoptosis, and sedative effects on aging-related diseases [9]. Senescence-accelerated mouse prone 8 (SAMP8) mice have been widely used in preclinical drug screening for improving cognitive function and revealing the mechanisms of intervention by traditional Chinese and western medicine in CI caused by aging [12,13].

Hippocampal pyroptosis activated by chronic inflammation reportedly plays an important role in the development of CI. In mice, plasma levels and hippocampal protein expression of cytokines such as NLR family pyrin domain containing 3 (NLRP3), cysteine-dependent aspartate-specific programmed-1 (caspase-1), interleukin-1*β* (IL-1*β*), interleukin-6 (IL-6), interleukin-18 (IL-18), and tumor necrosis factor-*β* (TNF-*β*) have revealed the potential roles of drugs in the management of pyroptosis and chronic inflammation [14]. Few studies have also shown that increased expression of caspase-1, IL-1*β*, IL-6, and IL-18 is associated with hippocampal pyroptosis in AD [15]. Therefore, the present study was designed to identify changes in pyroptosis in SAMP8 CI model mice treated with SCPE and to explore the effects of SCPE on the levels of NLRP3, ASC, caspase-1, GSDM-D, IL-1*β*, IL-6, IL-18, and *β*-amyloid (A*β*) expression in hippocampal tissues.

## 2. Materials and Methods

### 2.1. Material

SCPE is a famous traditional Chinese medicine composed of four Chinese herbs, *Acori graminei rhizoma* (*Acorus tatarinowii*), *Polygala tenuifolia* (*Polygala tenuifolia* Willd), tortoise shell (*Chinemys reevesii* (Gray)), and fossil fragments (*Fossilia Ossia Mastodi*). SCPE pills were provided by Beijing Tongrentang Co., Ltd., China. SCPE specimens were deposited in the synthesis laboratory of the College of Basic Medical Sciences, Heilongjiang University of Chinese medicine, Harbin, China. The pills were suspended in sterile water before being administered orally at a concentration of 0.1 g/mL and dosages of 2.3 g/kg/day, 4.6 g/kg/day, and 9.2 g/kg/day. These oral dosages for mice were determined in the preexperiment.

### 2.2. Animal Models

Seven-month-old 40 male SAMP8 and 10 male senescence-resistant mouse R1 (SAMR1) mice with a body weight of 20 ± 2 g were provided by the experimental animal center of the first affiliated hospital of Tianjin University of Chinese Medicine (animal license number: SCXK (Tianjin) 2015-0003). All of these experimental animals were raised in the drug safety evaluation center of Heilongjiang University of Chinese medicine with an SPF grade environment at a temperature (*T*) between 20 and 22°C, relative humidity between 40% and 60%, and a 12 h day/night cycle (6 : 00 am to 6 : 00 PM). All animals were kept for adaptive feeding for 7 days before the formal experiment during which they were free to eat (sterile feed) and drink (autoclaved water). The experimental protocol was approved by the Animal Care and Use Committee of Heilongjiang University of Chinese Medicine and abided by laboratory animal use regulations.

### 2.3. Groups and Administration

Forty SAMP8 mice were assigned to four groups: model group (model, mice were orally administered sterile water at 10 ml/kg/day for 28 consecutive days, *n* = 10), SCPE low-dose (LD) group (the mice were orally administered SCPE at 2.3 g/kg/day for 28 consecutive days, *n* = 10), SCPE medium-dose (MD) group (the mice were orally administered SCPE at 4.6 g/kg/day for 28 consecutive days, *n* = 10), and SCPE high-dose (HD) group (the mice were orally administered SCPE at 9.2 g/kg/day for 28 consecutive days, *n* = 10). Ten SAMR1 mice were assigned to a control group (control, mice were orally administered sterile water at 10 ml/kg/day for 28 consecutive days, *n* = 10). Water maze training was conducted on SAMR1 and SAMP8 mice 7 days before the start of administration to select mice with CI for formal experiments.

Morris water maze evaluation was conducted at different timepoints during the administration to evaluate the learning and memory ability of each group of mice. After the drug intervention ended, the mice were sacrificed, and samples of hippocampal tissue and serum were collected for further evaluation. The serum levels of IL-1*β*, IL-6, IL-18, and TNF-*β* were detected with enzyme-linked immunosorbent assay (ELISA). The expression levels of NLRP3, ASC, caspase-1, GSDM-D, IL-1*β*, IL-6, IL-18, and A*β* were detected by western blotting.

### 2.4. Morris Water Maze Was Used to Evaluate Learning and Memory Ability

Morris water maze training was conducted 7 days before drug administration, and 40 SAMP8 mice with CI were selected for formal experiments. According to previous studies and the preexperiment, the criterion for selecting mice with CI was a significantly prolonged escape latency (>80 s) compared with the control group [12,13]. Morris water maze evaluation was conducted on each group of mice at four time points: 7 days, 14 days, 21 days, and 28 days during administration. The water maze device in our laboratory is composed of a black circular pool with a diameter of 200 cm and a depth of 80 cm and divided into four quadrants with the same size. In the center of each quadrant wall above the water, 1 eye-catching color mark and 4 different colors were posted (Biobserve, Bonn, Germany). A black circular platform with a diameter of 10 cm was hidden 2 cm below the water surface and placed in the center of the third quadrant with the same position throughout the experiment. References around the pool (outside the water maze) included the experimenter position, which remained the same. This test was mainly divided into two aspects: (1) navigation experiment used to measure the learning ability of mice in the water maze, and the main evaluation index was escape latency (seconds) and (2) space exploration experiment used to measure the ability of mice to maintain long-term memory, and the main evaluation index was the number of platform crossings (times). Each mouse was separately evaluated in the experiment, and after each water maze evaluation, the dirty water was replaced, and the animal's fur was dried with an animal hair dryer according to animal welfare guidelines.

### 2.5. Sample Preparation

Mice were anesthetized with a small animal anesthesia machine (Shanghai Sango Biotechnology Co., Ltd., Shanghai, China) (isoflurane gas anesthesia) 24 h after the end of the final Morris water maze experiment (i.e., 29 days after the start of administration). Then, the blood of the mice was collected from the orbital vein and placed in a 10 mL biochemical tube. Centrifugation was carried out at room temperature at a rotational speed of 3300 RPM/min for 10∼15 min. The isolated serum was placed into a 2 mL cryopreservation tube that was autoclaved and placed in liquid nitrogen for rapid freezing and preservation. After the venous blood was taken, the mice were decapitated. Hippocampal tissue was rapidly removed with sterile tweezers and placed in 4°C refrigerated 0.9% saline for washing. Next, the tissue was placed on the prepared cold plate, and bilateral hippocampal tissue was rapidly dissected. Half of the hippocampus was fixed in 4% paraformaldehyde and embedded in paraffin for histological analysis. The other half of the hippocampus was stored in a 2 mL cryopreservation tube, which was rapidly frozen and stored in liquid nitrogen for western blot analysis.

### 2.6. Neuropathological Staining

Neuropathological changes were determined by hematoxylin-eosin (HE) staining, Congo red staining, and toluidine blue staining. Hippocampal tissue was preserved in 10% buffered formaldehyde, dehydrated by ethanol, cleared by xylene, embedded in paraffin, and cut into 4–6 mm slices. Sectioning was followed by xylene dewaxing, HE staining, Congo red staining, and toluidine blue staining, dehydration, and clearing. Finally, the sections were sealed, observed under a light microscope and photographed. Histopathological evaluation was detected by an expert pathologist with a microscope at 200x, 400x, and 800x. All stained slides were scanned with an APERIO automatic digital pathological section scanner (Nikon, Beijing, China), and representative images were recorded. Briefly, in each group, HE staining was mainly used to observe the pathological changes in hippocampal CA1 and CA3 regions. Congo red staining focused on the deposition of amyloid. Toluidine blue staining mainly reflects the shape and number of Nissl bodies in hippocampal neurons.

### 2.7. TUNEL Staining

After the experiment, paraffin sections were placed into xylene for dewaxing for 10 min. Next, the sections were moved to fresh xylene, followed by dewaxing for 5 min, 5 min anhydrous ethanol, and then 90% ethanol, 70% ethanol, and distilled water for 2 min each. Proteinase K was added, followed by digestion for 15 min. The sections were washed twice with PBS. Then, TUNEL detection liquid was added at 37°C and incubated in the dark for 60 min. The cells were washed with PBS liquid 3 times. Next, drops of POD were added at 37°C for 30 min in the wet box, followed by three washes with PBS. Then, DAB substrate was added for 5–10 min at room temperature, and slides were analyzed under a microscope. Hematoxylin staining, alcohol dehydration, xylene clearing, resin sealing, microscope observation, and photography were performed. The Image-Pro Plus 6.0 pathological image analysis system counted the number of positive nuclei, and each slice was observed with 6 fields of view. The average value of the 10 calculation results yielded the final number of TUNEL-positive nuclei.

### 2.8. Serum Inflammatory Factor Analysis

An IL-1*β*, IL-6, IL-18, and TNF-*β* cartridge was used in double-antibody sandwich ELISA (NanJing JianCheng Bioengineering Institute, Nanjing, China). Microwells were precoated with antibodies, followed by the addition of specimens, standards, and HRP-labeled detection antibodies for incubation. The wells were thoroughly washed and stained with the substrate TMB; TMB is transformed into blue by peroxidase catalysis and converted to the final yellow color under the activity of an acid. The depth of the color is positively correlated with the amount of inflammatory factors in the sample. The absorbance (OD value) was measured with a microplate reader at a wavelength of 450 nm to calculate the sample concentration.

### 2.9. Western Blot Analysis of Hippocampal Tissue

A portion of hippocampal tissue was placed in a frozen storage tube and immediately stored in liquid nitrogen for detection by western blotting. The total protein and nuclear protein were extracted from myocardial tissue. SDS-PAGE electrophoresis was performed with 50 *μ*g protein. After electrophoresis, the protein was transferred to the NC membrane. Five percent skimmed milk powder was added for blocking at room temperature for 1 h. Next, 5% BSA-diluted antibody was added for incubation at 4°C overnight. The film was washed with TBST 3 times for 15 min each. Next, secondary antibodies in diluent (1 : 1000) were incubated at room temperature for 1 h. Then, the film was washed with TBST 3 times for 15 min each. ECL chemiluminescence color rendering was used, and the film was exposed in a Bio-Rad device (Thermo Fisher Scientific Inc., Waltham, MA, USA) to detect NLRP3, ASC, caspase-1, GSDM-D, IL-1*β*, IL-6, IL-18, and A*β* protein expression (Cell Signaling Technology, Danvers, MA, USA).

## 3. Statistical Analyses

All experimental data are presented as the mean ± SD by SPSS 22.0 statistical software. Statistical significance was assessed by one-way ANOVA, and the paired *t*-test was used to examine the significance of the differences in Morris water maze results. *β* < 0.05 was regarded as statistically significant.

## 4. Results

### 4.1. Effects of SCPE on the Learning and Memory Ability of SAMP8 Mice

**Figure 1:**
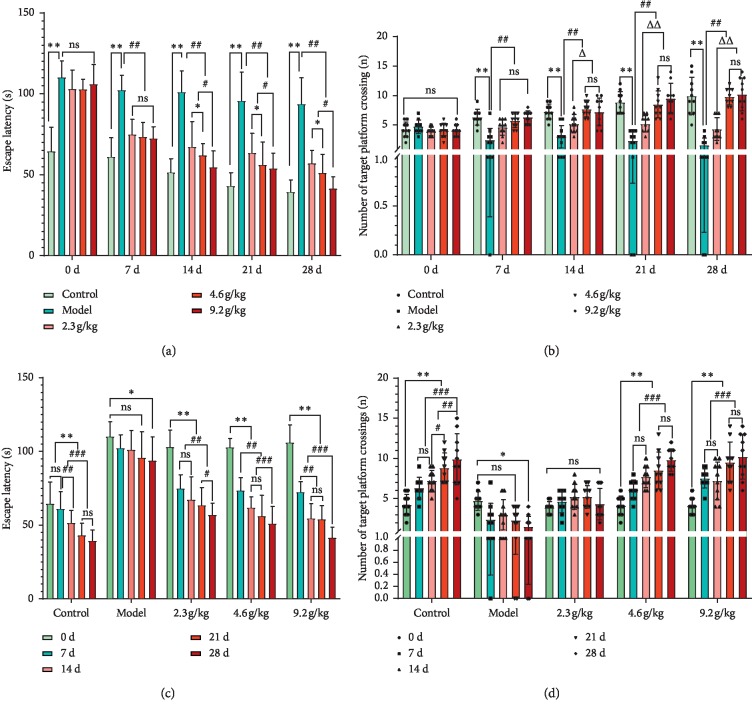
Learning and memory ability after the administration of SCPE. (a) Dose-effect relationship of the navigation experiment. (b) Dose-effect relationship of the space exploration experiment. (c) Time-effect relationship of the navigation experiment. (d) Time-effect relationship of the space exploration experiment. The data are shown as the mean ± SD of 10 mice per group; ^*∗∗*^versus the control group, *P* < 0.01 (a, b); ^##^versus the model group, *P* < 0.01 (a, b); ^*∗*^versus the low-dose group, *P* < 0.05 (a); ^#^versus the low- and medium-dose groups, *P* < 0.05 (a); ^Δ^versus the low-dose group, *P* < 0.05 (b); ^ΔΔ^versus the low-dose group, *P* < 0.01 (b); ^*∗∗*^versus the day 0, *P* < 0.01 (c, d); ^*∗*^versus the day 0, *P* < 0.05 (c, d); ^##^versus the 7^th^ day, *P* < 0.01 (c, d); ^###^versus the 28^th^ day, *P* < 0.01 (c, d). Ns: not significant.

As shown in Figure 1, compared with the control group (SAMR1 mice) before administration (0 days), the model group (SAMP8 mice) showed significantly prolonged escape latency, but the number of platform crossings was not significantly different (*P* > 0.05). These findings indicated that the 7-month-old SAMP8 mice used in this study had mild CI and did not reach the condition of severe dementia. After 7 days of administration, compared with the control group, the model group and LD/MD/HD groups showed a significant decrease in learning and memory ability (*P* < 0.01), and improvement in learning and memory ability by SCPE was not obvious. After 14 days of administration, SCPE significantly improved learning and memory (*P* < 0.01). SCPE also improved the learning and memory ability of mice at 21 days and 28 days after administration, and this ability was positively correlated with the dose and time (*P* < 0.01).

### 4.2. Effects of SCPE on Hippocampal Neuropathological Changes in SAMP8 Mice

#### 4.2.1. HE Staining Results

**Figure 2:**
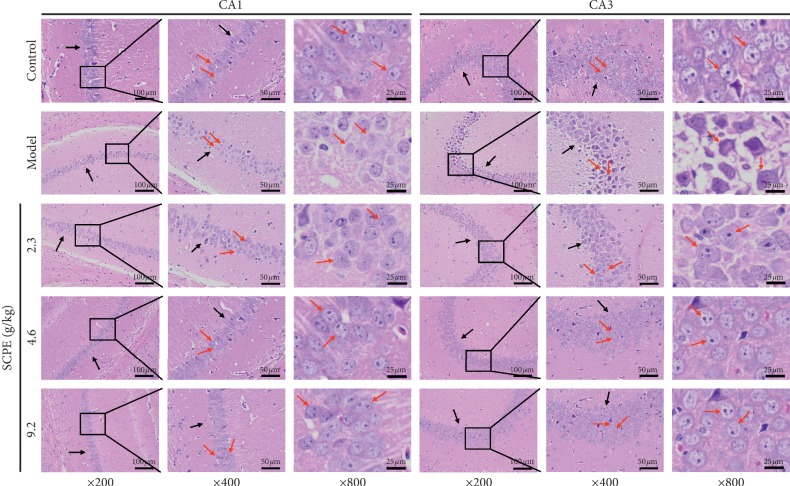
HE staining results of hippocampal CA1 and CA3 regions in each group. The black arrows show the arrangement and intercellular space of pyramidal cells in the hippocampal CA1 and CA3 regions. The red arrows show the typical morphology of neurons in the hippocampal CA1 and CA3 regions.

As shown in Figure 2, the control group neurons in hippocampal CA1 and CA3 regions were relatively complete and clear, with pyramidal cells arranged in a compact and tidy manner in uniform distributions (black arrow). The nucleosome in pyramidal cells exhibited a larger and rounder shape in the center of the nucleus (red arrow). In the model group, the number of neurons in hippocampal CA1 and CA3 regions was significantly reduced, and abnormal pathological changes such as degeneration and necrosis were observed. The size of pyramidal cells was generally reduced, and the shape was irregular (red arrow). Additionally, the arrangement of pyramidal cells was disordered and discontinuous (black arrow).

After different doses of SCPE, compared with the model group, the LD group showed pyramidal cells in hippocampal CA1 and CA3 regions that were shrunken but exhibited a less disordered (red arrow) and discontinuous arrangement and increased intercellular space (black arrow), accompanied by substantial numbers of degenerating necrotic neurons (red arrow). In the MD group, pyramidal cells in hippocampal CA1 and CA3 regions increased in size, with a relatively orderly (red arrow) and continuous arrangement (black arrow), smaller intercellular space, less nuclear shrinkage, and fewer degenerating necrotic neurons (red arrow). In the HD group, pyramidal cells in hippocampal CA1 and CA3 regions were arranged in an orderly manner; were clear and complete; showed normal morphology and structure (black arrow); and were evenly distributed with large and obvious nucleoli and abundant cytoplasm, accompanied by a small amount of nuclear condensation and a clear nuclear membrane (red arrow).

#### 4.2.2. Results of Congo Red Staining

**Figure 3:**
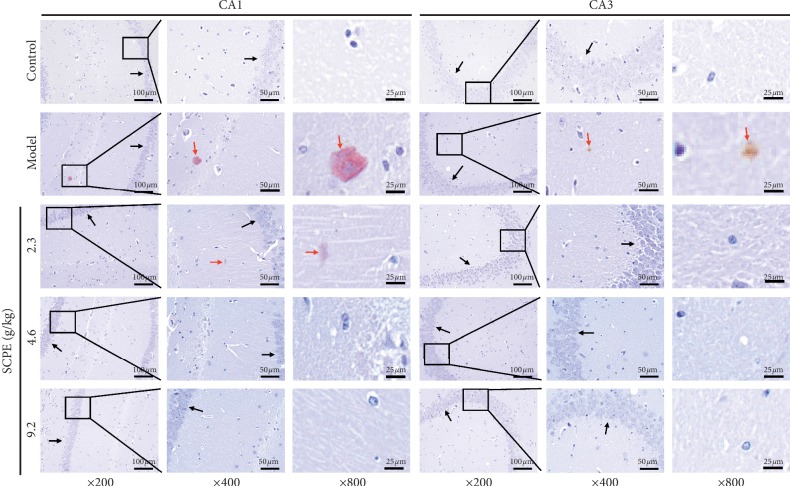
Congo red staining results of hippocampal CA1 and CA3 regions in each group. The black arrows show the arrangement and density of pyramidal cells in the hippocampal CA1 and CA3 regions. The red arrows show brick-red plaques in the hippocampal CA1 and CA3 regions.

As shown in Figure 3, Congo red staining in hippocampal CA1 and CA3 regions was negative, and there were no brick-red plaques or other abnormal changes in the control group (black arrow). In the model group, brick-red plaques caused by a large amount of scattered amyloid deposition were observed in hippocampal CA1 and CA3 areas (red arrow), with relatively small cell volumes and low densities (black arrow). In the LD group, a certain number of brick-red plaques was seen in hippocampal CA1 (red arrow), but the number was significantly reduced compared with that in the model group, with a relatively large number of shrinking cell volume and sparse arrangement (black arrow). In the MD and HD groups, there were no brick-red plaques in the hippocampal CA1 and CA3 areas. With normal cell volume and compact arrangement, the HD group showed significant pathological improvement compared with the model group (black arrow).

#### 4.2.3. Results of Toluidine Blue Staining

**Figure 4:**
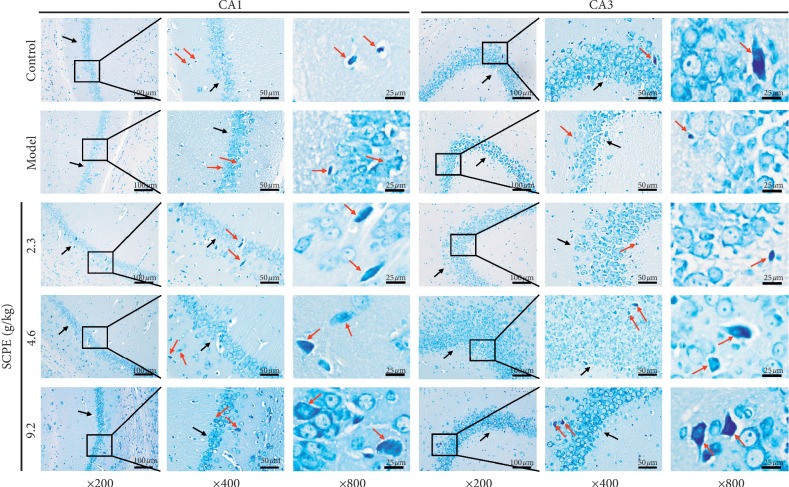
Toluidine blue staining results of hippocampal CA1 and CA3 regions in each group. The black arrows show the basic shape of the neurons in the hippocampal CA1 and CA3 regions. The red arrows show Nissl bodies in the hippocampal CA1 and CA3 regions.

As shown in Figure 4, Nissl bodies in the hippocampal CA1 and CA3 regions appeared as blue granules or plaques (red arrow). In the control group, neurons in hippocampal CA1 and CA3 regions had abundant Nissl bodies. In the model group, the number of Nissl bodies in hippocampal CA1 and CA3 regions was decreased or presented as Nissl body granulation (red arrow); Nissl bodies even disintegrated and disappeared (red arrow). In each SCPE group (LD, MD, and HD), the neurons in hippocampal CA1 and CA3 regions showed a granular distribution of Nissl bodies, and a small number of Nissl bodies exhibited scattered distribution (red arrow). The expression in the CA3 area was more obvious (red arrow), indicating that SCPE can improve the metabolism level of neurons in the hippocampus of SAMP8 mice. The basic shape of the neuron is indicated by a black arrow in each figure.

### 4.3. Effects of SCPE on the Cell Death of Hippocampal Neurons in SAMP8 Mice

**Figure 5:**
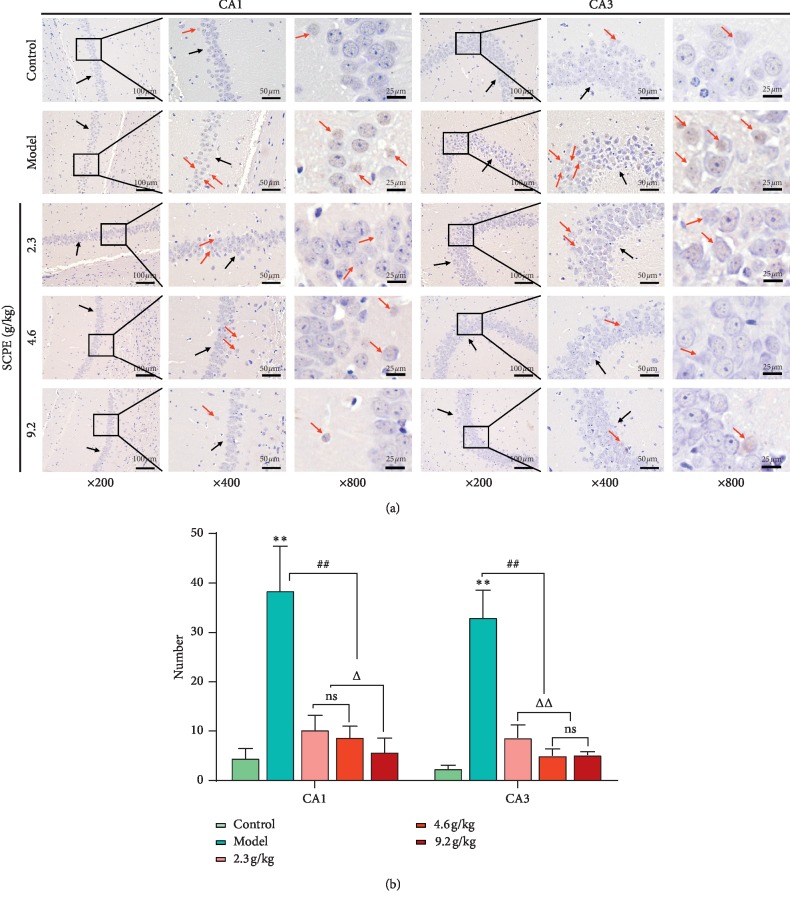
TUNEL staining results of hippocampal CA1 and CA3 regions in each group. (a) TUNEL staining morphology dead hippocampal neurons. The black arrows show the basic shape of the neurons. The red arrows show the TUNEL-positive cells. (b) Number of TUNEL-positive pyroptotic cells. The data are shown as the mean ± SD of 10 mice per group; ^*∗∗*^versus the control group, *P* < 0.01; ^##^versus the model group, *P* < 0.01; ^Δ^versus the low-dose group, *P* < 0.05; ^ΔΔ^versus the low-dose group, *P* < 0.01. Ns: not significant.

In the TUNEL staining results in Figure 5, TUNEL-positive cells are shown in dark brown (red arrow) and the basic shape of the neurons is indicated by a black arrow in each figure. Compared with the control group, the model group showed a significant increase in the number of TUNEL-positive cells in the hippocampal CA1 and CA3 regions (^*∗∗*^*P* < 0.01). Compared with the model group, the SCPE LD, MD, and HD groups showed a significant reduction in the number of TUNEL-positive cells in the hippocampus (^##^*P* < 0.01). Compared with the LD group, the MD group showed a significant decrease in the number of CA1 region TUNEL-positive cells (^Δ^*P* < 0.05), while the HD group showed a decrease in the number of CA1 and CA3 region TUNEL-positive cells (^Δ^*P* < 0.05, ^ΔΔ^*P* < 0.01). Compared with the MD group, the HD group showed a decrease in the number of CA1 TUNEL-positive cells (^Δ^*P* < 0.05).

### 4.4. SCPE Treatment Decreased the Levels of Serum Inflammatory Factors in SAMP8 Mice

**Figure 6:**
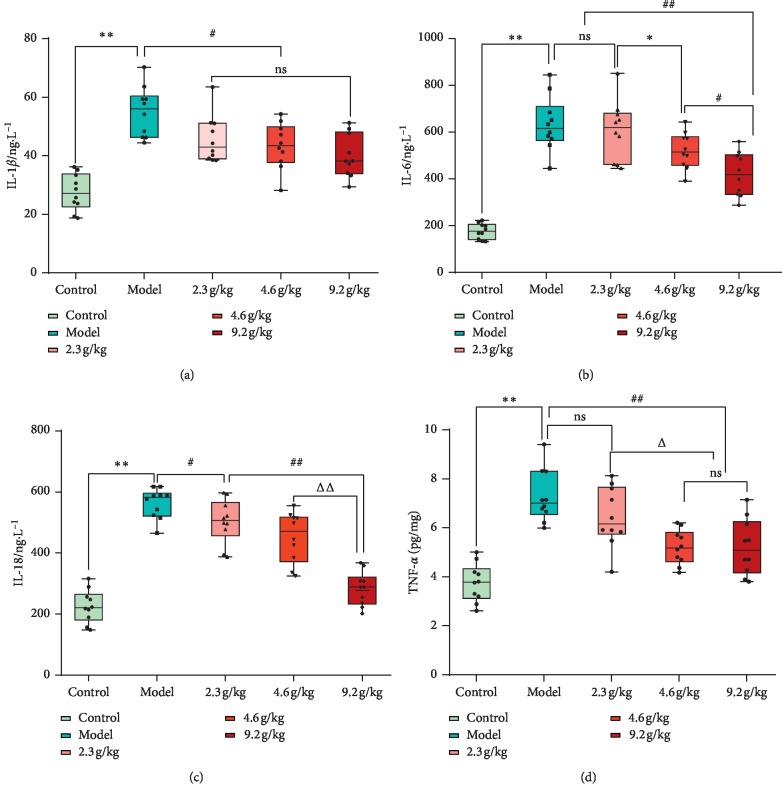
Level of serum inflammatory factors in SAMP8 mice treated with SCPE. (a) IL-1*β*. (b) IL-6. (c) IL-18. (d) TNF-*α*. The data are shown as mean ± SD of 10 mice per group; ^*∗∗*^, versus the control group, *P* < 0.01; ^#^versus the model group, *P* < 0.05 (a, c); ^##^versus the model group, *P* < 0.01 (b, d); ^*∗*^versus the low-dose group, *P* < 0.05; ^##^versus the low-dose group, *P* < 0.05 (c); ^Δ^versus the low-dose group, *P* < 0.05; ^#^versus the medium-dose group, *P* < 0.05 (b); ^ΔΔ^versus the medium-dose group, *P* < 0.01. Ns: not significant.

As shown in Figure 6, the serum levels of inflammatory factors IL-1*β*, IL-6, IL-18, and TNF-*α* in the model group were significantly higher than those in the control group (*P* < 0.01). Compared with the model group, the SCPE groups exhibited reduced levels of inflammatory factors IL-1 *β*, IL-6, IL-18, and TNF-*α* in the serum of mice after 28 d of SCPE administration, and the HD group showed the most obvious effect. The changes in IL-6 and IL-18 levels were strongly correlated with the dose of SCPE. This result suggested that SCPE inhibits the chronic inflammatory cascade reactions in SAMP8 mice.

### 4.5. SCPE Treatment Decreased NLRP3/Caspase-1 Signaling Pathway Expression in SAMP8 Mice

**Figure 7:**
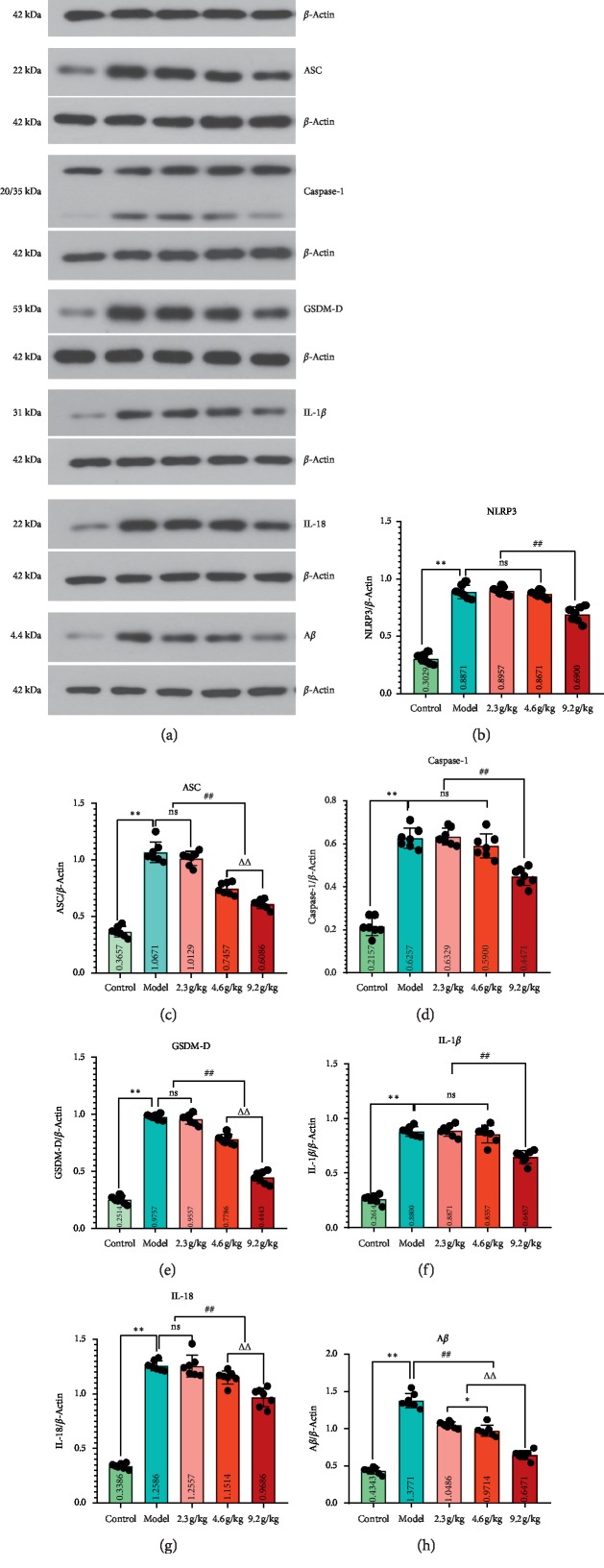
Protein expression of the NLRP3/caspase-1 signaling pathway after the administration of SCPE. (a) Expression of (b) NLRP3 protein, (c) ASC protein, (d) caspase-1 protein, (e) GSDM-D protein, (f) IL-1*β* protein, (g) IL-18 protein, and (h) A*β* protein in the hippocampus of each group. The data are shown as the mean ± SD of 7 mice per group; ^*∗∗*^versus the control group, *P* < 0.01; ^##^versus the model group, *P* < 0.01; ^*∗*^versus the low-dose group, *P* < 0.05; ^ΔΔ^versus the medium-dose group, *P* < 0.01. Ns: not significant.

As shown in Figure 7, the expression levels of NLRP3, ASC, caspase-1, GSDM-D, IL-1*β*, IL-18, and A*β* in the hippocampus in the model group were significantly higher than those in the control group (*P* < 0.01). After consecutive administration of SCPE for 28 days, the expression levels of NLRP3, ASC, caspase-1, GSDM-D, IL-1*β*, IL-18, and A*β* proteins in hippocampal tissue were downregulated to varying degrees, shown as follows.

As shown in Figures 7(b), 7(d), and 7(f), compared with the control group, the model group showed significantly increased expression levels of NLRP3, caspase-1, and IL-1*β* proteins in the hippocampus (^*∗∗*^*P* < 0.01). Compared with the model group, the LD and MD SCPE groups showed no significant changes in the expression levels of NLRP3, caspase-1, and IL-1*β* in the hippocampus (*P* > 0.05). Compared with the model group, the HD group showed significantly decreased expression levels of NLRP3, caspase-1m and IL-1*β* in the hippocampus (^##^*P* < 0.01).

As shown in Figures 7(c), 7(e), and 7(g), compared with the control group, the model group showed a significant increase in the expression levels of ASC, GSDM-D, and IL-18 protein in the hippocampus (^*∗∗*^*P* < 0.01). Compared with the model group, the LD SCPE group exhibited no significant change in the expression levels of ASC, GSDM-D, and IL-18 protein in the hippocampus (*P* > 0.05). Moreover, compared with the model group, the MD and HD SCPE groups showed significantly decreased expression levels of ASC, GSDM-D, and IL-18 protein in the hippocampus (^##^*P* < 0.01). In addition, compared with the MD group, the HD group exhibited a significant decrease in the expression levels of ASC, GSDM-D, and IL-18 protein in the hippocampus (^ΔΔ^*P* < 0.01).

As shown in Figure 7(h), the expression level of A*β* protein in the hippocampus in the model group was significantly increased compared with that in the control group (^*∗∗*^*P* < 0.01). Compared with the model group, the LD, MD, and HD groups exhibited significantly decreased hippocampal A*β* protein expression levels (^##^*P* < 0.01). The reduction in hippocampal A*β* protein expression was more pronounced in the HD group than in the MD group (^ΔΔ^*P* < 0.01).

## 5. Discussion

Mild CI caused by aging is an important early stage of dementia. As the degree of CI is relatively mild, it is easily overlooked by patients and medical workers in the clinic [16]. SAMP8 mice age rapidly and have been widely used in the study of the pathological mechanism of cognitive dysfunction and the screening of drugs to improve cognitive function for many years. These mice represent an ideal animal model to simulate the CI caused by aging factors. We selected 7-month-old SAMP8 and SAMR1 mice with the same genetic background as the controls. The Morris water maze experiment was used for behavioral evaluation. Compared with SAMP8 over 8 months old selected by previous dementia studies [17], 7-month-old SAMP8 mice showed a downward trend in learning ability (navigation experiment), but the memory ability (space exploration experiment) remained at the same level as that of SAMR1 mice, and the difference was not statistically significant (*P* > 0.05). As the experimental period progressed, mice in the control group showed better learning and memory ability after repeated training, and the escape latency and the number of platform crossings were better than those in the other groups (*P* < 0.01). Mice in the model group gradually showed a process of transformation from mild CI to dementia and showed a significant decline in learning and memory ability (*P* < 0.01). SCPE had a good, dose-dependent effect on improving cognitive function (*P* < 0.05 or *P* < 0.01). However, notably, in the behavioral evaluation of mice in each group, SAMP8 mice often suffer from physical exhaustion due to aging, which seriously affects the escape latency and number of platform crossings. To avoid the influence of this factor on the behavioral evaluation results, we adopted a strict water maze experiment protocol. The water temperature was controlled at 20∼30°C in the experiment, and all mice were under the same experimental conditions to ensure that the inevitable influencing factors exerted the same amount of interference among each group. Moreover, the feces generated after the experiment were cleaned in a timely manner, and the water in the water maze equipment was changed regularly to avoid contamination, thereby minimizing the experimental error.

In the morphological study of cognitive disorders, neurons in the hippocampal CA1 and CA3 represent the region of focus. HE staining was mainly used to observe the basic pathological structure of hippocampal tissue, such as the ordered arrangement of pyramidal cells and the degree of neuronal edema, but it could not reflect the deposition of amyloid caused by the neural inflammatory cascade reaction. During the onset and progression of mild CI to dementia, the chronic neural inflammatory cascade reaction plays a crucial role. Chronic inflammatory cascades in the hippocampus can lead to deposition of A*β* over time, and Congo red staining can reflect typical pathological changes [18]. Nissl bodies represent an important characteristic structure to evaluate the metabolism of neurons. Nissl bodies are mainly composed of free ribosomes and rough endoplasmic reticulum. The main chemical components of Nissl bodies have strong affinity for toluidine blue dye. Toluidine blue staining is the most commonly used method to display Nissl body morphology in neurons [19]. Under normal circumstances, Nissl bodies have relatively complete cell morphology, presenting light blue staining under the microscope with good definition. When pathological damage to neurons occurs, the metabolism level of neurons decreases and the Nissl bodies dissolve and disappear. In this part of the experimental study, we used HE staining, Congo red staining, and toluidine blue staining to observe the pathological changes in neurons in each group. Compared with the control group, the model group showed basic pathological damage in neuron number and pyramidal cell arrangement, volume, and shape. Moreover, a small amount of scattered amyloidosis in the hippocampal area of the model group revealed brick-red plaques, with relatively small cell volumes and low densities. In addition, the number of Nissl bodies in the hippocampal area of model mice decreased or presented as degranulation and disintegration or simply disappeared. SCPE can effectively improve the basic pathological changes in SAMP8 mice, including the deposition of amyloid protein, the number of Nissl bodies, and the amount of degranulation.

After the experiment, the effect of SCPE on the body's chronic inflammatory cascade reaction was investigated by detecting the levels of IL-1*β*, IL-6, IL-18, and TNF-*α* in serum. IL-1*β* is generally considered a subtype of IL-1, which is often found in the body as a nonactive precursor of IL-1 (pro-interleukin-1, pro-IL-1) under normal conditions. IL-1*β* can be released by cleavage of caspase-1 to promote chronic inflammation. IL-1*β* can be produced by a variety of cells (e.g., macrophages, endothelial cells, and vascular smooth muscle cells) and a variety of cytokines in the body. In the senescence-induced mild CI model, inflammatory factors such as IL-1*β* can activate endothelial cells and leukocytes in the body and brain tissue. IL-1*β* induces the adhesion of leukocytes and endothelial cells, leading to the aggravation of inflammatory necrosis of neurons in brain tissue and the decline in cognitive function [20]. IL-6 is a cytokine produced by activated T lymphocytes and fibroblasts that is involved in the growth and differentiation of tissue cells, inflammatory stress, etc. IL-6 is also known as B-lymphocyte cytokine and often induces B-cell proliferation and differentiation to produce antibodies. In addition, cytotoxic T lymphocytes can be activated to enhance the response capacity to chronic inflammation in the body and regulate the immune function of cerebral tissues [21]. IL-18 is also an important inflammatory factor that is often widely distributed in the hippocampus, striatum, and other brain regions and can promote the proliferation of T cells, induce the immune response of nerve cells, and activate and secrete inflammatory factors such as IL-1*β* and TNF-*α* [22]. TNF-*α* is an inflammatory factor produced by neutrophils, NK cells, macrophages, etc.; this factor can promote the entry of white blood cells into cerebrospinal fluid and participate in aggravating brain tissue inflammation and injury as well as exacerbating neuroimmune inflammation [23]. Aging is a major factor leading to chronic inflammatory responses in the brain tissue of SAMP8 mice and plays an important role in the pathogenesis of CI. Compared with the control group, the model group showed a significant increase in all inflammatory factors (*P* < 0.01). By contrast, SCPE reduced 4 of these inflammatory mediators in vivo in a dose-dependent manner. The HD group showed the best effect in reducing the chronic inflammatory response.

According to previous studies [24], chronic inflammation in cognitively impaired mice is closely related to the activation of NLRP3 inflammasome-dependent pyroptosis of neurons. Martinon et al. [25] first proposed the concept of the inflammasome in 2002. The inflammasome is mainly composed of effector protein caspase-1, apoptosis-related speckle-like protein (ASC), and cytoplasmic pattern recognition receptor (PRR). Inflammasomes recognize pathogen-associated molecular patterns (PAMPs) and host-derived damage-associated molecular patterns (DAMPs) to recruit and activate the proinflammatory protease caspase-1. Activated caspase-1 cleaves the precursors of IL-1*β* and IL-18, releasing large amounts of cytokines. Pyroptosis can be directly triggered by the activation of inflammasomes, and pyroptosis is a strongly proinflammatory process of programmed cell death. In most cases, inflammatory cascade reactions occur in the early stages of inflammation in brain tissue. Pyroptosis is an important type of cell death induced by initial inflammation in brain tissue during the progression of mild CI to dementia. The classic pyroptosis pathway is mediated by the NLRP3/caspase-1 signaling pathway. Based on studies in recent years, the NLRP3 inflammasome, NLRP1 inflammasome, AIM2 inflammasome, and IPAF inflammasome are the top four important inflammasomes in the process of pyroptosis. The NLRP3 inflammasome is considered an important molecular switch in the regulation of inflammation and plays an important role in the field of natural immunity. GSDM-D (Gasdermin-D) is the substrate of caspase-1/4/5/11 [26]. Under the action of various inflammasomes, the activation of caspase-1 can not only induce IL-1*β* and IL-18 to mature IL-1*β* and IL-18 but also leads to the transfer of the GSDM-D amino terminus (Gasdermin D-NT) of GSDM-D decomposition to the cell membrane to form active pores, allowing water molecules and other substances to enter the cell, leading to the swelling and lysis of cells [14]. GSDM-D is a key downstream factor of caspase-1. Therefore, inflammasome NLRP3, ASC, caspase-1, GSDM-D, IL-1*β*, and IL-18 are key signaling molecules for inflammation and cell pyroptosis and are involved in the process of CI and other cognitive disorders.

According to previous studies [27], the continuous activation of NLRP3 inflammasomes is involved in the deposition process of amyloid protein in CI and aging. Reversing the overexpression of NLRP3 inflammasomes can significantly reduce the incidence of pyroptosis and thus improve cognitive dysfunction induced by aging and other factors. Current research has shown that probenecid, as an inhibitor of the pannexin 1 (PANX1) channel, inhibits extracellular ATP release and reduces the levels of TNF-*α*, IL-6, and IL-1*β* in the hippocampus to effectively inhibit cell pyroptosis and improve cognitive function [28]. Pyroptosis has also been found in the hippocampal tissues of SAMP8 mice, and electroacupuncture can inhibit pyroptosis of the hippocampus by reducing IL-1*β*, NLRP3, ASC, and caspase-1 protein expression [29]. Another study also reported that aged mice treated with MCC950 (NLRP3 inflammasome inhibitor) could provide neuroprotective effects against pyroptosis by activating the inflammasome, thus reducing cognitive impairment. MCC950 could be an effective and potential drug for elderly CI patients [24].

The focus of our present study was to determine whether it is possible to reveal the pathological mechanism of mild CI caused by aging and the pharmacological effect of SCPE intervention in the early stage of dementia accompanied by chronic inflammatory reactions through the classical NLRP3/caspase-1 pyroptosis signaling pathway. Based on the TUNEL staining results, compared with the control group, the model group showed a significant increase in the number of TUNEL-positive cells and apoptotic bodies were widely distributed in the tissues of the hippocampal CA1 and CA3 regions. After the SCPE intervention, the number of TUNEL-positive cells in SAMP8 mice was effectively reduced, especially in the HD group. Western blotting was performed on the classic NLRP3/caspase-1 signaling pathway-related proteins and A*β* proteins. Compared with the control group, the model group exhibited significantly higher expression of NLRP3, ASC, caspase-1, GSDM-D, IL-1*β*, IL-18, and A*β* in the hippocampus, while SCPE could effectively reduce the expression of the NLRP3, ASC, caspase-1, GSDM-D, IL-1*β*, IL-18, and A*β* proteins.

In summary, we showed that SCPE may improve the brain pathological changes, amyloid deposition, and neuron metabolism level of SAMP8 mice by reducing A*β* deposition and slowing down the classic NLRP3/caspase-1-mediated pyroptosis pathway of the neuroimmune inflammatory cascade, thus playing a pharmacological role in improving the cognitive function of mice with CI.

## Data Availability

The data used to support the findings of this study are available from the corresponding author upon request.
